# Discovery of Novel HPK1 Inhibitors Through Structure-Based Virtual Screening

**DOI:** 10.3389/fphar.2022.850855

**Published:** 2022-03-14

**Authors:** Huizhen Ge, Lizeng Peng, Zhou Sun, Huanxiang Liu, Yulin Shen, Xiaojun Yao

**Affiliations:** ^1^ College of Chemistry and Chemical Engineering, Lanzhou University, Lanzhou, China; ^2^ Institute of Agro-Food Science and Technology Shandong Academy of Agricultural Sciences, Key Laboratory of Agro-Products Processing Technology of Shandong Province, Key Laboratory of Novel Food Resources Processing Ministry of Agriculture, Jinan, China; ^3^ Academy of Advanced Interdisciplinary Studies, Qilu University of Technology (Shandong Academy of Sciences), Jinan, China; ^4^ School of Pharmacy, Lanzhou University, Lanzhou, China; ^5^ Gansu Computing Center, Lanzhou, China

**Keywords:** HPK1 inhibitor, immunotherapy, virtual screening, molecular docking, molecular dynamics simulation

## Abstract

Hematopoietic progenitor kinase (HPK1) is a negative regulator of T-cell receptor and B-cell signaling, which has been recognized as a novel antitumor target for immunotherapy. In this work, Glide docking-based virtual screening and kinase inhibition assay were performed to identify novel HPK1 inhibitors. The kinase inhibition assay results demonstrated five compounds with IC_50_ values below 20 μM, and the most potent one (compound M074-2865) had an IC_50_ value of 2.93 ± 0.09 μM. Molecular dynamics (MD) simulations were performed to delve into the interaction of sunitinib and the identified compound M074-2865 with the kinase domain of HPK1. The five compounds identified in this work could be considered promising hit compounds for further development of HPK1 inhibitors for immunotherapy.

## Introduction

Immunotherapy has become an effective treatment for some types of cancer (Gamat and McNeel, 2017; Rolfo et al., 2017; Kono et al., 2018; Park et al., 2018; Greten et al., 2019; Sunami and Kleeff, 2019; He and Xu, 2020). Immune checkpoint inhibitors that target the cytotoxic T-lymphocyte antigen-4 (CTLA-4) and the programmed cell death protein 1 (PD-1) pathways resulted in strong and durable responses in a series of tumors (Farkona et al., 2016; Ribas and Wolchok, 2018). For patients with tumor metastasis, immunotherapy is expected to be the most effective form of treatment (Restifo et al., 2016). Therefore, it is an urgent need to find effective drugs for immunotherapy.

Hematopoietic progenitor kinase (HPK1) is a member of a family of mitogen-activated protein kinase kinase kinase kinase (MAP4K) of Ste20 serine/threonine kinases, which is predominantly expressed in hematopoietic cells and has been shown to be a negative immune regulator of T-cell receptor (TCR) and B-cell signaling (Alzabin et al., 2009; Sawasdikosol et al., 2012; Sawasdikosol and Burakoff, 2020). The HPK1 signaling pathway was mainly studied in T cells (Hernandez et al., 2018; Sawasdikosol et al., 2020; Si et al., 2020). Active HPK1 kinase phosphorylates serine residue 376 of the adaptor protein Src homology 2 domain containing leukocyte protein of 76 kDa (SLP76), which binds to negative regulator complex 14-3-3 (Sauer et al., 2001; Di Bartolo et al., 2007). This results in subsequent ubiquitin-induced, proteasome-mediated degradation of SLP-76 and blocks downstream kinase signaling required for initiation and proliferation of T cells, suppressing an innate immune response (Lasserre et al., 2011). T cells from HPK1-deficient mice (Shui et al., 2007) and HPK1 kinase-dead mice (Hernandez et al., 2018; Liu et al., 2019) showed enhanced proliferation in response to TCR stimulation compared to wild-type mice. In addition, in syngeneic tumor models MC38 and GL261 (glioma), kinase-dead mice exhibited enhanced antitumor immunity, irrespective of whether or not anti-programmed death-ligand-1 (anti-PD-L1) exists (Sawasdikosol et al., 2007; Hernandez et al., 2018). Importantly, compared with mice lacking other negative regulators (e.g., CTLA-4), HPK1 knockout mice and HPK1 kinase-dead mice did not show fatal inflammation (Degnan et al., 2021). These data indicate the potential role of small-molecule HPK1 inhibitors in cancer treatment.

Recently, several classes of HPK1 inhibitors have been discovered (Supplementary Figure 1). Sunitinib, a multi-receptor tyrosine kinase (RTK) inhibitor, was measured, and the inhibition constant (K_i_) against WT HPK1 kinase *in vitro* was found to be about 10 nM by Johnson et al. (2019). Wu et al. (2019) reported GNE-1858 as an HPK1 inhibitor with potent inhibitory effects (IC_50_ = 1.9 nM). Yu et al. (2021) identified piperazine analog XHS as a potent HPK1 inhibitor with an IC_50_ value of 2.6 nM against HPK1 kinase and an IC_50_ value of 0.6 μM in the SLP76 peripheral blood mononuclear cell (SLP76 PBMC) assay. Vara et al. (2021) reported diaminopyrimidine carboxamide compound 22 with an IC_50_ value of 0.061 nM. BMS company conducted high-throughput screening followed by lead optimization and finally obtained compound K as an HPK1 inhibitor (IC_50_ = 2.6 nM). This compound is a potent HPK1 inhibitor, with 50-fold greater selectivity than the MAP4K family (Degnan et al., 2021; You et al., 2021). Sabnis (2021) reported substituted exomethylene-oxindole compound C17 (IC_50_ = 0.05 nM) as a novel HPK1 inhibitor. So far, two companies have shared information about their clinical trials for HPK1 inhibitors. Tradwell reported the initiation of a phase 1/2 study to evaluate the safety and tolerability of CFI-402,411 with advanced solid malignancies (Clinicaltrials.gov, 2021). This research was launched in August 2020. The second company, Beigene, launched a study to investigate the safety, pharmacokinetics, tolerability, and preliminary antitumor activity of HPK1 inhibitor BGB-15025 in patients with advanced solid tumors (Clinicaltrials.gov, 2020). The structures of CFI-402411 and BGB-15025 have not been disclosed. Although the aforementioned studies have made some meaningful progress, only a few drugs (e.g., CFI-402411 and BGB-15025) currently in clinical trials use HPK1 inhibitors as an immunotherapy strategy. Therefore, it is urgent to find novel and structurally diverse HPK1 inhibitors with immunotherapy efficacy.

Structure-based virtual screening using molecular docking has faster computational speed and lower cost than the experimental screening based on large databases of entity compounds and has become a powerful tool for the discovery of hit compounds (Xu et al., 2014; Shen et al., 2015; Wang et al., 2018; Khair et al., 2019; Wang et al., 2020; Wu et al., 2020; Zhang et al., 2020; Opo et al., 2021; Sahihi et al., 2021). In this work, we conducted the Glide docking-based virtual screening and *in vitro* kinase inhibition assay to discover potent compounds targeting HPK1 kinase. About 1.5 million molecules from the ChemDiv database (version 2019) were used for virtual screening. The kinase inhibition assay results indicated that five compounds with potent inhibitory activity were discovered in our work. The most potent one (compound M074-2865) shows an IC_50_ value of 2.93 ± 0.09 μM. Then, molecular dynamics (MD) simulations and binding free energy calculations were conducted to explore the detailed information of the kinetic mechanism between inhibitors (sunitinib and newly identified compound M074-2865) and HPK1 kinase domain.

## Materials and Methods

### Preparation of Receptor and Ligands

The X-ray crystal structure of human HPK1 kinase in complex with sunitinib (PDB ID: 6NFY; resolution: 2.17Å) (Johnson et al., 2019) was derived from the Protein Data Bank (https://www.rcsb.org/). Chain B in the structure is more complete than chain A, so chain B in the structure is retained. The protein preparation wizard panel in the work of Schrödinger (2015) was used to add hydrogen, eliminate all water molecules, assign charges and protonation states at pH 7.0, and minimize the structure with the OPLS-2005 force field (Kaminski et al., 2001). The prepared chain B structure was taken to generate a grid using the *Glide* 6.6 module in the work of Schrödinger (2015). Sunitinib was selected as the center of the grid, and the grid box was defined as a 20 × 20 × 20Å. About 1.5 million molecules in the ChemDiv (version 2019) database were used as the screening source. All compounds were imported into the *Ligprep* 3.3 module in the work of Schrödinger (2015) to prepare ligands. The ligand preparation process is as follows: the ionization states of molecules at pH 7.0 ± 2.0 were generated with *Epik* 3.1 (Shelley et al., 2007), stereoisomers were generated, and one low-energy conformation was generated per ligand.

### Structure-Based Virtual Screening

The generated grid and prepared ligands were then subjected to docking-based virtual screening using *Glide 6.6.* The prepared ligands were filtered by applying Lipinski’s rule of five, including 1) hydrogen bond donor <5, 2) hydrogen bond acceptor <10, 3) predicted octanol/water partition coefficient (QPlogPo/w) <5, 4) rotatable bond <10, and 5) molecular weight <500 (Lipinski, 2004), and the ligands with reactive functional groups were removed. Glide screening is divided into three levels: 1) Glide/HTVS was used for rapid high-throughput screening, and the 10% top ranked molecules were retained for the next screening. 2) Then, Glide SP was performed and the 10% top ranked molecules were retained. 3) Finally, we used Glide XP to screen ligands and retained the top 4,000 molecules to select potential inhibitors. The Prime MM-GBSA method in Maestro was used to calculate the binding free energy of these 4,000 molecules to HPK1 kinase. Then, we used the k-means clustering protocol in Canvas 2.3 to cluster these 4,000 molecules into 100 groups. By visual inspection of the binding modes of HPK1 kinase–ligand, 39 compounds were selected and purchased from Topscience Co., Ltd. (https://www.tsbiochem.com) for the kinase inhibitory activity assay.

### *In Vitro* Kinase Inhibition Assay

HPK1 kinase protein was purchased from Invitrogen (Cat. No. PV6357), and kinase substrate25 was purchased from GL (Cat. No. 117881). First, the Caliper mobility shift assay (Caliper MSA) (Card et al., 2009; Pollack et al., 2011; Wang et al., 2015) was used to evaluate the inhibitory activity of the 39 compounds against HPK1 kinase at a concentration of 50 μM. Then, active compounds (inhibition >60% at concentration of 50 μM) were selected to determine the IC_50_ values. Sunitinib (Johnson et al., 2019; Wang et al., 2021), purchased from Bide Pharmatech Ltd., was evaluated as a positive control compound. 250 nL of the tested compound in 100% DMSO (eight concentrations, ranged from 6 nM to 100 μM) was dispersed in a 384-well plate, and 250 nL of 100% DMSO was added to negative and positive control wells, respectively. In addition, the initial concentration of sunitinib was 1 μM, and 10 concentrations (ranged from 0.0508 nM to 1 μM) were detected in duplicate. Then, 10 μL of enzyme solution was added to the compound and positive control wells, respectively, and 1 × kinase buffer was added to the negative control wells. The mixture was centrifuged at 1,000 rpm for 30 s and then mixed and incubated at room temperature for 10 min. 15 μL of 25/15 × ATP and kinase substrate25 in the 1 × kinase buffer were added to each well. The plate was incubated at room temperature for 2 h. Finally, 30 μL terminated buffer was added to terminate the kinase reaction. Conversion rate data were read with Caliper EZ ReaderⅡ. The IC_50_ values of concentration-dependent curves were fitted by GraphPad Prism 6.0.

### Molecular Dynamics Simulation

To investigate the binding mechanism of inhibitors to HPK1 kinase, the MD simulations were conducted on the HPK1 kinase in complex with sunitinib and the identified representative active compound (M074-2865). The initial structure of the HPK1–sunitinib complex was derived from the Protein Data Bank. The initial structure of HPK1–M074-2865 for MD simulation is obtained from Glide docking. The restrained electrostatic potential (RESP) (Bayly et al., 1993; Batterman et al., 2014) calculated by the Hartree–Fock (HF) method with a 6-31G* basis set in the Gaussian09 package (Frisch et al., 2009) was used to fit the partial charges for the inhibitors. The ff14SB force field (Maier et al., 2015) and the general AMBER force field (gaff) (Wang et al., 2003) were used for HPK1 kinase and the inhibitors, respectively. The complex was solvated in TIP3PBOX at a distance of 12 Å to the boundary. After adding chloride ions to neutralize the systems, the systems were minimized, heated, and equilibrated. 500 ns MD simulations were performed at 300 K with 1.0 atmospheric pressure in an NPT ensemble. The trajectory analysis was performed *via* the *cpptraj* module in AMBER18 (Case et al., 2005).

### Binding Free Energy Calculation

The binding free energy (ΔG_bind_) of inhibitors (sunitinib and compound M074-2865) to HPK1 kinase was analyzed by the Molecular Mechanics Generalized Born Surface Area (MM-GBSA) method (Hou et al., 2010; Liu et al., 2010; Xu et al., 2013). The binding free energy was calculated as follows:

$$\Delta {\text{G}}_{\text{bind}}=G_{complex}-\left(G_{receptor}+G_{ligand}\right)$$ (1)

$$G_{bind}=\Delta H-T\Delta S=E_{gas}+G_{sol}-T\Delta S$$ (2)

$$E_{gas}=E_{\mathrm{int}}+E_{ele}+E_{vdw}$$ (3)

$$G_{sol}=G_{GB}+G_{nonpl,sol}$$ (4)

$$G_{nonpl,sol}=\gamma *SASA$$ (5)

1,000 snapshots extracted from the last 20 ns trajectories of the system were used to calculate binding free energy. The residue free energy decomposition identifies the key residues around the inhibitor by decomposing the total free energy into each residue without considering the contribution of entropy. Only 50 of the 1,000 snapshots were used to evaluate the entropy term (−TΔS) due to the large amount of computational cost and the long computational time.

## Results and Discussion

### Structure-Based Virtual Screening

**FIGURE 1:**
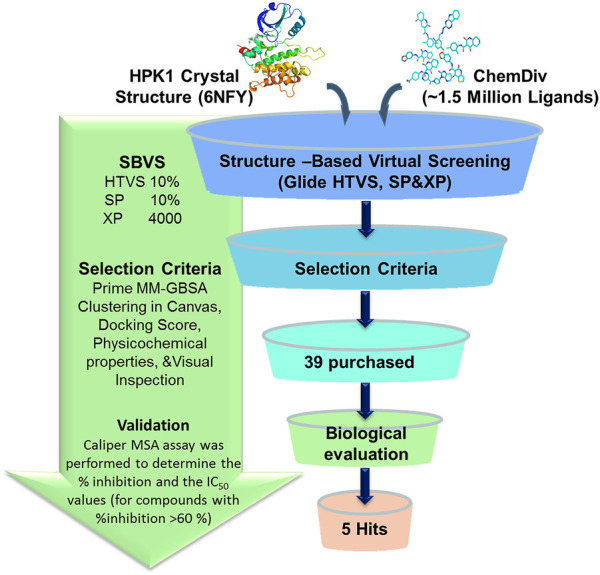
Flowchart of structure-based virtual screening and *in vitro* kinase inhibition assay for HPK1 inhibitor.

The workflow of the structure-based virtual screening is shown in Figure 1. Through three-level (HTVS, SP, XP) screenings, the compounds with the top 4,000 Glide docking scores were obtained. According to binary fingerprints, the 4,000 compounds were clustered into 100 classes using k-means clustering in Canvas 2.3. The selection of each class of compounds follows the following principles: 1) lower docking score; 2) lower MM/GBSA score; 3) lower molecular weight; 4) forming hydrogen bond interaction with the residues Glu92 and Cys94; and 5) occupying the binding pocket of a suitable size molecule. Based on these, 39 compounds were purchased for evaluation of the HPK1 kinase inhibition assay. The molecular structures of the 39 selected compounds are shown in Supplementary Figure 2. The physical characteristics and docking results of the 39 selected compounds are summarized in Supplementary Table 1.

### *In Vitro* Kinase Inhibition Assay

**FIGURE 2:**
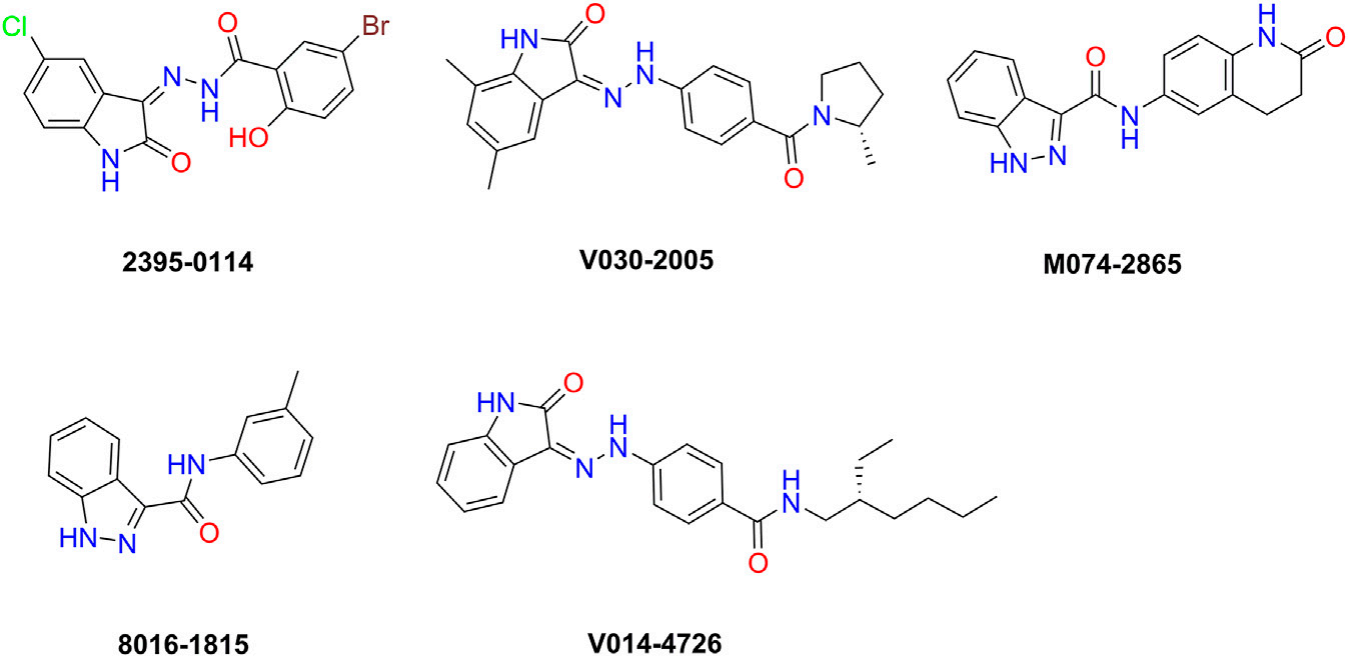
Chemical structures of the five compounds identified by structure-based virtual screening and kinase inhibition assay.

**FIGURE 3:**
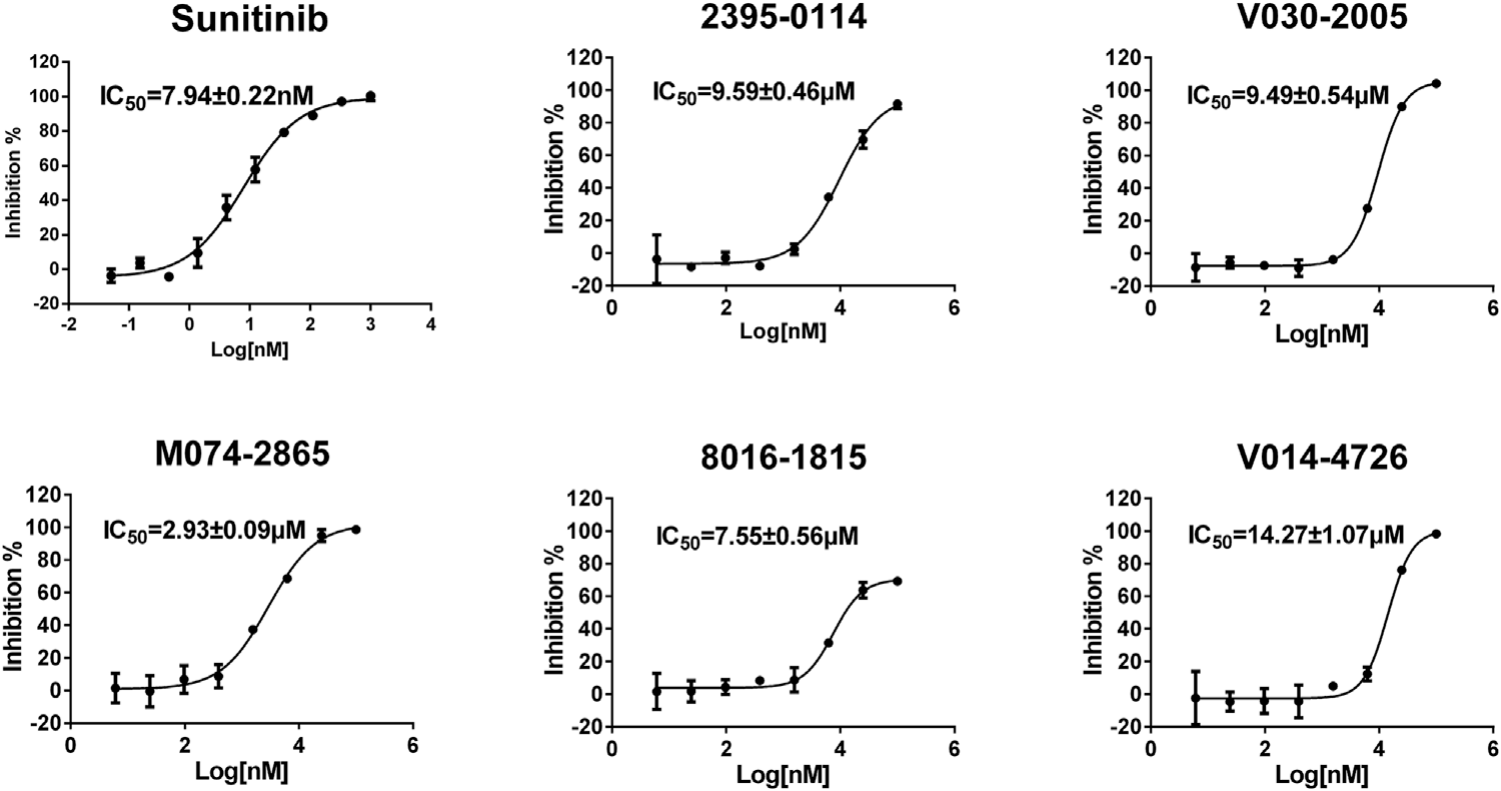
Inhibitory activity curves of sunitinib (positive control compound) and the five active compounds against HPK1 kinase.

To evaluate the inhibitory effect of screened HPK1 inhibitors, the kinase inhibitory activity *in vitro* was determined by the Caliper MSA method. Five compounds (compound 2395-0114, V030-2005, M074-2865, 8016-1815, and V014-4726) exhibited over 60% inhibition at 50 μM. Next, the IC_50_ values of these five compounds were determined. As summarized in Table 1, the IC_50_ values of the five compounds ranged from 2.93 ± 0.09 μM to 14.27 ± 1.07 μM. The chemical structures of the five compounds are depicted in Figure 2. The inhibitory activity curves of sunitinib (positive control compound) and five active compounds against HPK1 kinase are depicted in Figure 3. These five compounds have the potential to become promising lead compounds for further optimization.

**TABLE 1:** XP docking scores, MM-GBSA energy, physicochemical properties, and HPK1 inhibition activities of the screened compounds.

Compd	Docking score	Prime MM-GBSA	QPlogPo/w^a^	mol MW^b^	PSA^c^	Inhibition (%)50 μM	IC_50_(μM)
2395-0114	−9.66	−69.70	3.70	394.61	90.79	93.50 ± 0.60	9.59 ± 0.46
V030-2005	−9.02	−76.33	3.85	376.46	73.80	92.40 ± 1.00	9.49 ± 0.54
M074-2865	−9.19	−68.15	2.14	306.32	108.05	90.40 ± 0.80	2.93 ± 0.09
8016-1815	−8.96	−63.72	3.13	251.29	57.78	73.10 ± 0.10	7.55 ± 0.56
V014-4726	−9.55	−85.13	4.60	392.50	82.59	71.50 ± 2.80	14.27 ± 1.07

a Predicted octanol/water partition coefficient.

b Molecular weight.

c Polar surface area. All experiments are performed in duplicate. Data are expressed as means ± standard deviation (SD).

### Binding Mode Prediction

**FIGURE 4:**
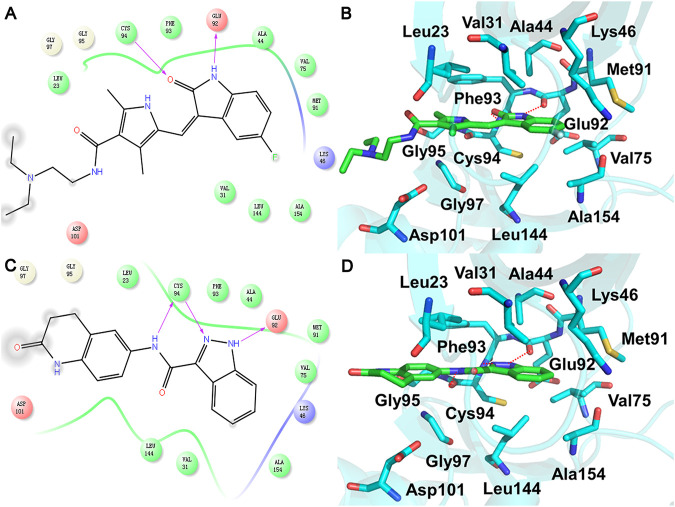
2D model diagram of HPK1 with **(A)** sunitinib and **(C)** compound M074-2865 and the 3D binding mode of HPK1 with **(B)** sunitinib and **(D)** M074-2865. The inhibitors are shown as green sticks, and the key residues of HPK1 kinase binding to inhibitors are shown as cyan sticks. The hydrogen bond between HPK1 kinase and inhibitors is shown as a red dash.

In order to clarify the HPK1 inhibitor binding mode, the docking poses generated by Glide XP were analyzed. As shown in Figure 4A and Figure 4B, the crystal structure of HPK1 bound to sunitinib (PDB ID: 6NFY) demonstrated that the 1) oxindole scaffold is surrounded by residues Val31, Ala44, Lys46, Val75, Glu92, Phe93, Cys94, Leu144, and Ala154; one hydrogen bond (H-bond) is formed between the NH of the oxindole and the backbone carbonyl of Glu92, and another H-bond is formed between the backbone NH of Cys94 and the carbonyl of the oxindole, 2) 3,5-dimethyl-1H-pyrrol-2-yl surrounded by residues Leu23, Phe93, Gly95, Gly97, and Asp101 is located at the binding pocket entrance, 3) {[2-(diethylamino) ethyl] amino} carbonyl is exposed to the solvent, and 4) the F atom at position 5 of oxindole is surrounded by residues Lys46 and Ala154. The docked binding mode of ligand M074-2865 with HPK1 and the binding mode of X-ray crystal structure of sunitinib with HPK1 were compared. As shown in Figure 4C and Figure 4D, the docking structure of HPK1 with compound M074-2865 demonstrated that 1) the indazole scaffold is surrounded by residues Val31, Ala44, Lys46, Val75, Glu92, Phe93, Cys94, Leu144, and Ala154; one H-bond is formed between the NH of the indazole and the backbone carbonyl of Glu92, and another H-bond is formed between the backbone NH of Cys94 and the N of the indazole, 2) the amide group surrounded by residues Leu23, Val31, Phe93, and Cys94 is located at the binding pocket entrance, and an H-bond is formed between the NH of the amide and the backbone carbonyl of Cys94, and 3) 3, 4-dihydroquinolin-2 (1H)-one group is surrounded by residues Leu23, Gly95, Gly97, and Asp101, and {3, 4-dihydroquinolin-2 (1H)-one} carbonyl is exposed to the solvent. According to the analysis of the binding modes of the two inhibitors, compound M074-2865 shows a similar binding mode to sunitinib with HPK1 kinase. The similar binding mode can also be observed for the other four compounds (compound 2395-0114, V030-2005, 8016-1815, and V014-4726), according to the binding patterns predicted by the Glide docking (Supplementary Figure 3).

### Stability of the HPK1 Inhibitor System

**FIGURE 5:**
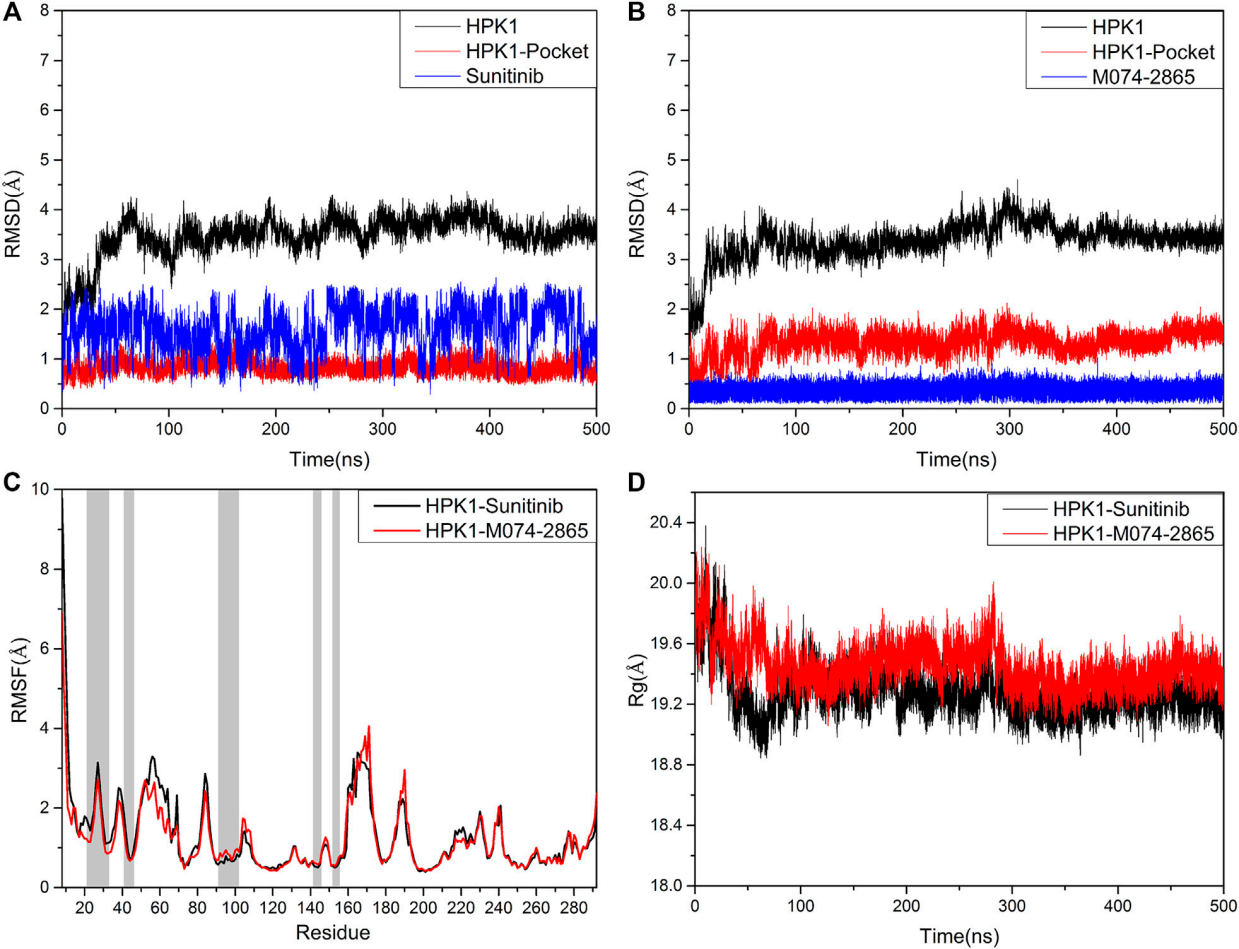
**(A)** RMSD of the backbone atoms of HPK1 kinase, the backbone atoms of HPK1 kinase pocket, and the heavy atoms of sunitinib. **(B)** RMSD of the backbone atoms of HPK1 kinase, the backbone atoms of HPK1 kinase pocket, and the heavy atoms of compound M074-2865. **(C)** RMSF values of HPK1 residue backbone atoms. **(D)** Mass-weighted radius of gyration.

The stability of each system was evaluated by measuring the RMSD of the backbone atoms of HPK1, the backbone atoms of HPK1 pocket, and the heavy atoms of ligand throughout the simulation period. As depicted in Figure 5A and Figure 5B, the RMSD plots showed that two systems reach a convergence after 100 ns. The RMSD of sunitinib showed higher amplitude than the identified inhibitor M074-2865. This indicates that compound M074-2865 can bind to HPK1 more stably than sunitinib. The simulation results show that the root-mean-square fluctuation (RMSF) values of the two systems have a similar trend, and the N-terminals of HPK1 protein fluctuate greatly (Figure 5C). As shown in Figure 5D, the gyration radii (Rg) of the two complexes were stable after 300 ns MD simulation, and the structure of the HPK1–sunitinib complex is about as compact as that of the HPK1–M074-2865 complex. Our analysis confirmed that each simulation system remained stable throughout the simulation.

### Hydrogen Bond Analysis

To monitor the formation of an H-bond between the inhibitors and HPK1 kinase during the MD simulations, hydrogen bond occupancy was calculated, as shown in Table 2. In the HPK1–sunitinib system, the stable H-bonds are formed between the NH of the oxindole and the backbone carbonyl of Glu92 (99.92% occupancy) and between the backbone NH of Cys94 and the carbonyl of the oxindole scaffold (99.74% occupancy). Compared with the Glide docking result (Figure 4), there are two additional H-bonds. The NH of 3,5-dimethyl-1H-pyrrol-2-yl formed an H-bond with the backbone carbonyl of Cys94 (13.74% occupancy), and the NH of {[2-(diethylamino) ethyl] amino} carbonyl formed an H-bond with the side chain carboxyl of Asp101 (11.46% occupancy). In the HPK1–M074-2865 system, the stable H-bonds are formed between the NH of the indazole and the backbone carbonyl of Glu92 (99.82% occupancy) and between the backbone NH of Cys94 and the N of the indazole scaffold (83.92% occupancy). The NH of the amide forms an unstable H-bond with the backbone carbonyl of Cys94 (18.35% occupancy). On the one hand, the H-bond between the carbonyl of the oxindole scaffold of sunitinib and the backbone NH of Cys94 (99.74%) is more stable than that between the N of the indazole scaffold of M074-2865 and the backbone NH of Cys94 (83.92%). On the other hand, there is an additional H-bond between the NH of {[2-(diethylamino) ethyl] amino} carbonyl and the side chain carboxyl of Asp101 (11.46% occupancy). The aforementioned information can explain the fact why sunitinib has better inhibition activity against HPK1 kinase than compound M074-2865.

**TABLE 2:** Analysis of hydrogen bond interaction between HPK1 and the two inhibitors (sunitinib and compound M074-2865).

Ligand	Acceptor	Donor	Occupancy (%)	Distance (Å)	Angle (°)
Sunitinib	GLU92@O	ligand@HN2	99.92	2.87	163.24
	ligand@O1	CYS94@HN	99.74	2.87	160.14
	CYS94@O	ligand@HN1	13.74	3.24	125.65
	ASP101@OD1	ligand@HN3	11.46	3.25	136.58
M074-2865	GLU92@O	ligand@HN2	99.82	2.90	164.61
	ligand@N1	CYS94@HN	83.92	3.13	145.82
	CYS94@O	ligand@HN4	18.35	3.25	129.66

### Binding Free Energy Calculation

The binding free energy (ΔG_bind_) was calculated by the MM-GBSA method to explore the binding difference between sunitinib and compound M074-2865 from the energy, as shown in Table 3. ΔG_bind_ for sunitinib and compound M074-2865 is −25.01 kcal/mol and −17.32 kcal/mol, respectively, which is consistent with the order of experimental IC_50_ values. The results also revealed that the non-polar interaction (−50.10 kcal/mol for sunitinib and −39.52 kcal/mol for compound M074-2865) was the main contributor to the interactions between inhibitors and HPK1, while the polar interaction (9.16 kcal/mol and 9.44 kcal/mol for sunitinib and compound M074-2865, respectively) was unfavorable for the binding. The main difference in ΔG_bind_ between sunitinib and compound M074-2865 is non-polar interactions (−50.10 kcal/mol for sunitinib and −39.52 kcal/mol for compound M074-2865).

**TABLE 3:** ΔG_bind_ for the two inhibitors (sunitinib and compound M074-2865) bound to HPK1 kinase by the MM-GBSA method (kcal/mol).

Energy	Sunitinib	M074-2865
ΔE_ele_	−23.84	−12.46
ΔE_vdw_	−44.47	−35.46
ΔE_gas_	−68.30	−47.92
ΔG_GB_	33.00	21.90
ΔG_nonpl,sol_	−5.63	−4.06
ΔG_sol_	27.38	17.84
ΔG_pl_	9.16	9.44
ΔG_nonpl_	−50.10	−39.52
ΔH (GB)	−40.93	−30.08
−TΔS	15.92	12.76
ΔG_bind_	−25.01	−17.32
IC_50_	7.94 ± 0.22 nM	2.93 ± 0.09 μM

**FIGURE 6:**
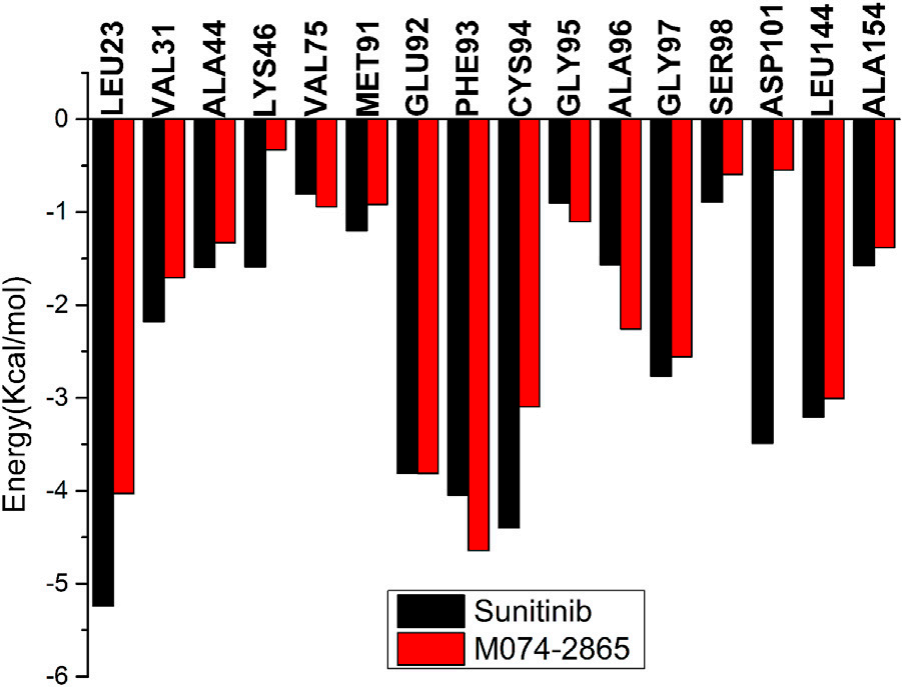
Total energy contribution comparison of HPK1 inhibitors binding to key residues.

**FIGURE 7:**
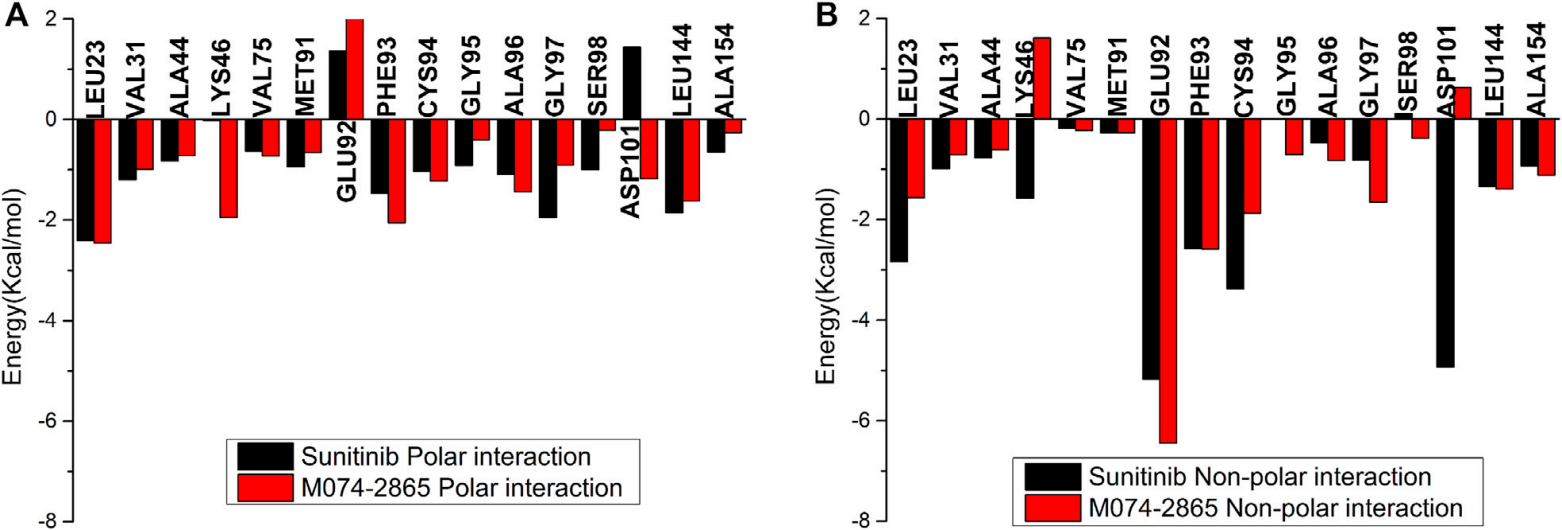
Polar energy contribution **(A)** and non-polar energy contribution **(B)** comparison of HPK1 inhibitors binding to key residues.

The binding free energies of HPK1 kinase–inhibitor complexes are decomposed to each residue to evaluate the detailed contributions of interacting residues in the binding of inhibitors to HPK1 kinase. The residues with energy contributions over 0.5 kcal/mol are listed in Figure 6. The residues with an energy contribution of more than 1.5 kcal/mol were considered to be critical; twelve key residues Leu23, Val31, Ala44, Lys46, Glu92, Phe93, Cys94, Ala96, Gly97, Asp101, Leu144, and Ala154 were identified in the HPK1–sunitinib complex. Key residues Leu23, Lys46, Cys94, and Asp101 were identified as the dominant difference for sunitinib and M074-2865 binding to HPK1 with absolute difference of energy between sunitinib and M074-2865 more than 1.0 kcal/mol. When sunitinib binds to HPK1, the energy contributions of dominant difference residues are Leu23: −5.24 kcal/mol, Lys46: −1.59 kcal/mol, Cys94: −4.40 kcal/mol, and Asp101: −3.49 kcal/mol. However, when the inhibitor was changed to M074-2865, this favorable free energy contribution is transformed into a relatively less favorable contribution due to Leu23 (−4.02 kcal/mol), Lys46 (−0.33 kcal/mol), Cys94 (−3.10 kcal/mol), and Asp101 (−0.55 kcal/mol). To further explore the reasons for the decreased energy contribution of the residues, we compared the polar contribution (sum of electrostatic and polar solvation interaction, as shown in Figure 7A) and non-polar contribution (sum of van der Waals and non-polar solvation, as shown in Figure 7B) of the aforementioned four key residues. The decrease in energy contribution of the four residues (Leu23, Lys46, Cys94, and Asp101) is mainly due to the loss of non-polar contribution of Leu23, Lys46, Cys94, and Asp101.

Hematopoietic progenitor kinase (HPK1), a negative regulator of T-cell receptor (TCR) and B-cell signaling, has been recognized as a novel antitumor immunotherapy target. HPK1 inhibitors block inhibitory signals that would reduce the ability of T cells to clear the tumor. So far, only few drugs (e.g., CFI-402411 and BGB-15025) in clinical trials have been used as HPK1 inhibitors for immunotherapy. In this study, by combining structure-based virtual screening and kinase inhibition assay, we discovered five inhibitors. The binding patterns predicted by the Glide docking show a similar trend. We further conducted molecular dynamics simulations on the HPK1 kinase in complex with sunitinib and the identified representative active compound M074-2865, and we found that compound M074-2865 had good inhibitory activity, mainly due to the hydrophobic interactions between compound M074-2865 and key residues (Leu23, Val31, Phe93, Ala96, Gly97, Leu144, and Ala154) and the hydrogen bond interaction between compound M074-2865 and the residues (Glu92 and Cys94). Meanwhile, the binding free energy and the entropic change were obtained by using MM-GBSA calculation and normal mode analysis, respectively. It was indicated that enthalpy was the main force driving interactions between HPK1 and inhibitors. On the basis of molecular docking, hydrogen bond analysis, and ΔGbind calculation results, we found the difference of binding mode between sunitinib and compound M074-2865 with HPK1 kinase is that the H-bond between the carbonyl of the oxindole scaffold of sunitinib and the backbone NH of Cys94 (99.74%) is more stable than that between the N of the indazole scaffold of M074-2865 and the backbone NH of Cys94 (83.92%), an additional H-bond between the NH of {[2-(diethylamino) ethyl] amino} carbonyl group and the side chain carboxyl of Asp101 (11.46% occupancy), and the reduction of energy contribution of the non-polar interaction of four residues Leu23, Lys46, Cys94, and Asp101. Therefore, the inhibitory activity may be improved by optimizing the structure of compound M074-2865 to enhance the hydrogen bond interaction with Cys94 and add the hydrogen bond interaction with Asp101 or increase the non-polar interaction with Leu23, Lys46, Cys94, and Asp101. We hope that this study would provide some guidance for virtual screening or design of novel HPK1 inhibitors.

## Conclusion

In this study, the Glide docking-based virtual screening and kinase inhibition assay were conducted to discover HPK1 inhibitors from the ChemDiv database. As a result, five compounds showed IC_50_ values in the range from 2.93 ± 0.09 μM to 14.27 ± 1.07 μM. MD simulations and binding free energy calculations showed that the non-polar interactions are the dominating force for the inhibitors binding to HPK1 kinase. The H-bonds between the inhibitors and the residues (Glu92, Cys94, and Asp101) play a critical role in inhibition activity. In brief, enhancing the hydrogen bond interactions (residues Glu92, Cys94, and Asp101) and non-polar interactions between HPK1 kinase and the inhibitors will contribute to further structural optimization. The five novel HPK1 inhibitors identified in this work can be used as the lead compounds for further optimization.

## Data Availability

The original contributions presented in the study are included in the article/Supplementary Material; further inquiries can be directed to the corresponding authors.

## Supplementary Material

Supplementary Figure 1 Chemical skeleton of the reported HPK1 inhibitors.

Supplementary Figure 2 Molecular structures of the 39 selected compounds.

Supplementary Figure 3 2D diagram of the binding mode of HPK1 with **(A)** compound 2395-0114, **(B)** compound V030-2005, **(C)** compound 8016-1815, and **(D)** compound V014-4726.

Supplementary Table 1 Physical characteristics and docking results of 39 selected compounds.
