# Supplementary material for: Discovery of Novel HPK1 Inhibitors Through Structure-Based Virtual Screening
Source: Front Pharmacol. 2022 Mar 14;13:850855. doi: 10.3389/fphar.2022.850855 (PMC8967249; doi:10.3389/fphar.2022.850855)
Supplement: Supplementary file 1 [file Table1.DOCX]

**Supplentary Information**

**Discovery of Novel HPK1 Inhibitor Through Structure-Based Virtual Screening**

**Table S1** Physical characteristics and docking results of 39 selected compounds

| IDNUMBER | ^a^donorHB | ^b^accptHB | ^c^QPlogPo/w | ^d^B_rotN | ^e^mol MW | ^f^PSA | docking score | Prime MM-GBSA |
| --- | --- | --- | --- | --- | --- | --- | --- | --- |
| 1927-8030 | 1 | 3.25 | 2.87 | 4 | 299.26 | 90.79 | -10.25 | -66.06 |
| 8004-1348 | 2 | 4.25 | 2.53 | 4 | 295.30 | 90.79 | -10.08 | -63.40 |
| 1348-0964 | 2 | 4.25 | 2.62 | 4 | 309.32 | 90.79 | -10.04 | -66.18 |
| 1986-3869 | 2 | 6 | 2.13 | 5 | 322.32 | 99.66 | -10.00 | -71.04 |
| P177-0143 | 2 | 3.5 | 3.01 | 4 | 323.37 | 93.93 | -9.86 | -71.86 |
| Y042-5744 | 2 | 5 | 2.62 | 1 | 309.32 | 76.24 | -9.83 | -65.02 |
| G824-0148 | 1 | 4.25 | 2.61 | 4 | 303.27 | 82.99 | -9.79 | -60.63 |
| 1348-0530 | 2 | 5.25 | 1.29 | 4 | 326.27 | 136.61 | -9.74 | -66.00 |
| 2395-0114 | 1 | 3.25 | 3.70 | 3 | 394.61 | 90.79 | -9.67 | -69.70 |
| 6540-0274 | 1 | 5.75 | 2.02 | 4 | 311.29 | 102.03 | -9.66 | -60.20 |
| 8004-1847 | 2 | 5.25 | 1.64 | 4 | 340.30 | 136.61 | -9.59 | -67.80 |
| V014-4726 | 2 | 6 | 4.60 | 9 | 392.50 | 82.59 | -9.55 | -80.61 |
| 1348-0491 | 2 | 4.25 | 2.52 | 2 | 360.17 | 90.79 | -9.55 | -66.08 |
| 1348-0492 | 2 | 4.25 | 2.52 | 1 | 360.17 | 90.79 | -9.47 | -68.50 |
| D330-1305 | 1 | 4 | 4.01 | 4 | 311.38 | 54.75 | -9.46 | -71.84 |
| D330-0475 | 1 | 3.25 | 3.61 | 3 | 281.35 | 50.44 | -9.42 | -70.58 |
| D330-0677 | 1 | 3.25 | 4.40 | 4 | 360.25 | 48.13 | -9.41 | -75.30 |
| D330-0144 | 1 | 3 | 3.22 | 2 | 263.30 | 51.95 | -9.32 | -64.56 |
| M074-2865 | 2 | 5 | 2.14 | 2 | 306.32 | 108.05 | -9.19 | -68.15 |
| Y041-3516 | 1 | 3.25 | 3.89 | 5 | 299.37 | 67.01 | -9.17 | -68.09 |
| Y043-4102 | 3 | 4.5 | 1.55 | 1 | 267.29 | 86.46 | -9.17 | -60.43 |
| K026-0262 | 1 | 4.75 | 1.84 | 3 | 323.26 | 121.03 | -9.16 | -69.81 |
| D330-0221 | 1 | 3.25 | 4.35 | 5 | 360.25 | 50.90 | -9.09 | -72.82 |
| 8017-3076 | 2 | 4.25 | 3.16 | 4 | 293.32 | 67.01 | -9.05 | -62.53 |
| Y020-9154 | 0 | 3 | 3.59 | 2 | 282.34 | 58.20 | -9.05 | -70.33 |
| V030-2005 | 1 | 6.5 | 3.85 | 3 | 376.46 | 73.80 | -9.02 | -76.33 |
| P163-0088 | 0 | 2.75 | 3.62 | 3 | 320.75 | 92.30 | -9.02 | -64.04 |
| S606-0886 | 1 | 6 | 2.36 | 2 | 314.36 | 85.66 | -8.99 | -72.07 |
| K844-1259 | 0 | 4.75 | 2.90 | 6 | 342.35 | 126.77 | -8.98 | -43.86 |
| P163-0117 | 0 | 4.25 | 2.48 | 3 | 330.31 | 111.01 | -8.98 | -60.94 |
| 8016-1815 | 1 | 2.5 | 3.13 | 2 | 251.29 | 57.78 | -8.97 | -63.73 |
| G491-0854 | 0 | 5 | 2.88 | 3 | 330.77 | 99.66 | -8.93 | -68.77 |
| K844-1246 | 0 | 2.75 | 3.92 | 4 | 312.37 | 89.18 | -8.86 | -38.06 |
| 1348-1372 | 3 | 6.25 | 2.24 | 4 | 338.76 | 99.08 | -8.85 | -62.93 |
| S606-0911 | 1 | 9 | 0.58 | 2 | 310.32 | 111.34 | -8.83 | -65.60 |
| 7062-0731 | 1 | 7 | 2.53 | 5 | 349.35 | 90.70 | -8.80 | -69.04 |
| P163-0707 | 0 | 3.5 | 3.49 | 6 | 330.36 | 101.55 | -8.74 | -52.27 |
| Y020-9071 | 0 | 3.75 | 2.99 | 3 | 284.31 | 67.43 | -8.74 | -72.75 |
| P684-0005 | 0 | 3.75 | 2.98 | 3 | 312.33 | 110.21 | -8.31 | -51.01 |

^a^Hydrogen bond donor. ^b^Hydrogen bond acceptor. ^c^Predicted octanol/water partition coefficient. ^d^rotatable bond. ^e^Molecular weight. ^f^Polar surface area.
